# Challenges and Opportunities in Designing and Deploying Remote Health Monitoring Technology for Older Adults With Cancer

**DOI:** 10.1093/geroni/igac057

**Published:** 2022-09-01

**Authors:** Virginia LeBaron

**Affiliations:** School of Nursing, University of Virginia, Charlottesville, Virginia, USA

**Keywords:** Cancer care, Oncology, Sensors, Smart health, Wireless health

## Abstract

Remote health monitoring (RHM) technologies (eg, wearables, smart phones, embedded sensors, and telehealth platforms) offer significant opportunities to improve health and wellness for older adults facing serious illness. This article highlights key challenges and opportunities for designing and deploying RHM systems in the context of caring for older adults with cancer, with an emphasis on the key role nurses can play in this work. Focal topics include user-centered design, interdisciplinary collaboration, addressing health inequities and disparities, privacy and data security, participant recruitment and burden, personalized and tailored care, rapid technological change, family caregiver perspectives, and naturalistic data collection. It is critical for nurses to be aware of both challenges and opportunities within each of these areas in order to develop RHM systems that are optimally beneficial for patients, family caregivers, clinicians, and organizations. By leveraging their unique knowledge of the illness experience from the patient, family, and health care provider perspective, nurses can make essential clinical and scientific contributions to advance the field of RHM.

**Translational Significance:** Remote health monitoring (RHM) offers tremendous potential to improve the health and wellness of older adults facing serious illnesses such as cancer. Nurses are essential partners to help inform the design and deployment of meaningful RHM systems but must be aware of key challenges and opportunities related to this technology.

Few of us will live our lives without being directly affected by cancer. As we age, we are statistically likely to either develop cancer ourselves (39% lifetime risk for American women; 40% for men ^[1]^) or become a caregiver for someone with cancer ^(2)^. As an illness experience, cancer is particularly complicated. The biological heterogeneity of the disease, lengthy and multimodal therapies (eg, surgery, chemotherapy, and radiation), unpredictable trajectories, serious stressors that affect all quality-of-life domains (eg, physical, emotional, social, spiritual, and financial), and rapidly evolving treatment options place challenging demands on patients, families, clinicians, health care systems and society at large. Effective tools and strategies are needed along the entire cancer care continuum―from prevention and screening, through active treatment to survivorship, and at the end-of-life―to support all different types of patients and families, each with their own unique contexts and needs.

While no single approach can address all of the challenges the disease of cancer presents, remote health monitoring (RHM) offers exciting possibilities to support patients, informal and professional caregivers, and organizations (^3,4^). RHM encompasses a broad range of technologies (such as wearable devices, telehealth platforms, smart phones, and environmental and biosensors) that can be deployed with diverse patient populations outside of the traditional acute care or clinic setting to collect health-related data that can inform care and even deliver interventions, often in real-time. Data can be gathered using passive and active approaches. Passive RHM requires little or no user engagement and can involve collecting ambient data (such as room temperature) from a home using environmental sensors, or measuring continuous physiological data, such as heartrate or motion, via a smartwatch. In contrast, active RHM data collection requires additional user engagement, often in the form of reporting some type of health-related information through a mobile app, telephone, virtual platform, or ecological momentary assessment (EMA), brief surveys delivered via a portable device. Some RHM systems rely exclusively on either passive or active data collection; others use a combination of both. Data collected by RHM are shared in multiple ways, most commonly with clinicians (and sometimes with patients and their caregivers) through a variety of different platforms (eg, web dashboards; direct linkages to electronic health records) to guide the patient’s plan of care or deliver automated just-in-time interventions. A key advantage of RHM is early detection of worsening or concerning symptoms, ideally preventing hospitalizations, emergency department visits, or even death ^(5)^. The exploding field of remote health has gained even more momentum in the wake of the COVID-19 pandemic as we have proven on a global scale that it is possible to provide efficient, high-quality health care remotely, and in some situations, it is the preferred method.

The purpose of this article is to highlight key challenges and opportunities for designing and deploying RHM systems in the context of caring for older adults with cancer, with an emphasis on the key role nurses can play in this work. While this article focuses on cancer-related RHM, the literature is replete with examples of RHM (many of them nurse-led) used to achieve health outcomes with diverse patient populations, including to reduce agitation in patients with dementia ^(6)^, monitor activity and glucose levels in diabetes ^(7)^, and promote self-care among community-dwelling older adults ^(8)^. Specific to cancer, RHM approaches have been used to monitor and manage posttreatment symptoms ^(9–13)^, provide real-time telehealth visits for patients with cancer in rural areas ^(14)^, and improve pain management (^15,16^), as just a few examples. A number of excellent literature reviews highlight the role nurses play in leading cancer-care interventions (^17,18^), more generally, and specifically related to RHM (^19,20^).

As the frontline care providers who spend the most time with patients, nurses are uniquely poised to inform the design and deployment of RHM systems. Nurses are adept and creative problem solvers, with training that prioritizes implementing practical solutions to help alleviate suffering. Importantly, nurses intimately understand the myriad challenges that patients and their family caregivers encounter as they navigate serious illness and the value of RHM to prevent or mitigate distress and debility. The input of nurses is critical to ensure RHM data collection are feasible and ethical, and that results are interpreted and utilized to develop interventions that are safe, actionable, and clinically relevant. Nurses also play a key role in facilitating patient uptake of RHM technology; for example, Wells (2022) specifically attributes the success of a remote health intervention designed to support patients taking oral anticancer therapy to the expertise of oncology nurse navigators who provided the critical link between patients, clinicians, and the technology ^(21)^. In short, nurses are experts at assessing and monitoring patients, and their expertise is essential in building technology that does the same.

## Discussion

Key topics related to RHM are discussed below, but some important caveats regarding scope are warranted. This article focuses on RHM in the context of cancer care, as it is a growing population of older adults with important health-related needs (and where my own research has focused). However, much, if not all, of what is discussed is generalizable and applies to other patient populations; certainly, key RHM issues such as privacy and equity are not pertinent to just patients with cancer. Additionally, the primary focus of this article is on how nurses can design robust systems to *collect* high-quality RHM data, but less about how RHM data are *used* to change or alter patient care. In other words, this is an article about challenges and opportunities related to remote health monitoring, not remote health management. The latter involves important and interesting discussions about how remote health data are processed, analyzed, and shared to develop interventions that can be deployed in real-time to improve health outcomes; these are critical topics, but beyond the scope of this article. It is also important to note that the line between remote health “monitoring” and “management” can be blurry. In our previous pilot work designed to monitor pain in patients with advanced cancer, we found that the monitoring process itself can serve as a form of intervention. For example, even though we were not delivering a traditional intervention related to a patient’s pain, our postdeployment evaluations revealed that simply monitoring pain with our sensor system improved communication and awareness of symptom management within dyads ^(22)^.

The list below is not intended to be exhaustive, nor an in-depth, “state of the science” discussion of each topic, but instead to provide an overview for nurse scientists or others considering or already engaged in similar research. It is also not intended to be a technical summary regarding specific computing and engineering aspects of RHM (although many such excellent articles exist [^5,23^)). When applicable, examples are provided from my research with the Behavioral and Environmental Sensing and Intervention for Cancer (BESI-C) (^15,24^) RHM system. Because of the reality that within each challenge lie opportunities, and within each opportunity lie related challenges, each topic is presented with a discussion of both related challenges and opportunities and instances of where these intersect. It is hoped that the following discussion will stimulate ideas and directions for future research and be a helpful reference for those exploring the field.

### Challenges and Opportunities

#### User-centered design

It is wrong to assume that older adults are unable or unwilling to use RHM, or that they will derive less benefit from such technology ^(25)^. However, older adult interest and use of RHM will be lower if platforms do not adequately account for expected developmental needs. For example, text font on wearables or smartphone apps should be as large as possible, or easily adjustable. Additional considerations may be needed for older patients with cancer, such as ensuring interfaces do not require fine or precise motor movements (like pressing small buttons), that may be difficult or impossible for patients suffering from neuropathy, a common side effect of cytotoxic therapy that causes numbness and tingling in the extremities. Utilizing a structured user-centered, participatory design process, as well as collaborating with human-factors experts (those that study how humans interact with technology), is essential in the development of RHM systems for older adults with cancer. The specifics of these design processes vary, but typically involve end-user surveys and interviews combined with beta-testing. We found structured interviews with patients and caregivers, combined with input from clinical partners, essential in validating the design features of system components, as well as confirming which variables to measure with BESI-C ^(24)^. Additionally, because nurses are particularly attuned to the patient experience, they can offer important insights into features of RHM systems that may be unnecessary, unduly burdensome, or simply inappropriate.

#### Interdisciplinary collaboration

Designing and deploying successful RHM systems is truly a team effort and requires a sustained commitment to interdisciplinary collaboration. Nurse investigators benefit from the expertise of engineers, data scientists, and biostatisticians, and likewise contribute their own unique knowledge and complementary skillset related to clinical relevance, study design, and methods for data collection and analysis (both qualitative and quantitative). Nurses may be surprised to learn how much they have in common with engineering colleagues, who share their interest and commitment to solving real-world problems that help people live better, safer lives ^(26)^. Although interdisciplinary collaboration is absolutely essential for nurses working in RHM, assembling functional and productive interdisciplinary teams is time consuming, and frankly, at times frustrating. Different disciplines have varying priorities, timelines, languages and professional expectations and norms related to communication, collaboration, study implementation, student engagement, funding/budgets, and dissemination of findings. It is often helpful to “test the waters” with lower-stake investments, such as with an abstract submission or small, intramural pilot funds, to assess the alignment and compatibility of interdisciplinary teams before embarking on larger projects.

#### Addressing health inequities and disparities

Perhaps most critically, RHM systems, if thoughtfully designed and equitably available, can address critical health inequities and disparities. Patient groups that have been historically marginalized and victims of systemic and structural racism and discrimination could receive more inclusive and respectful care if RHM is implemented well. For example, it is well documented that patients with cancer from underrepresented groups experience inadequate pain management ^(27–29)^. Imagine a scenario where robust RHM systems track and record the patient’s experience with pain, and longitudinal data are presented to the outpatient clinician in such a way that reduces bias and the risk of undertreatment of pain. For this to be successful, however, it is essential to understand how social determinants of health and cultural norms may affect the acceptability of perceived surveillance by RHM systems, and to design and deploy RHM systems that are sensitive to these concerns. One approach to help with this is to ensure diverse stakeholders are involved as collaborative partners in the early phases of system design and throughout implementation. Access to, and familiarity with, technology should not be the primary driver of an individual’s ability to benefit from RHM; both researchers and clinicians should be prepared to articulate how RHM systems they design and deploy help bridge―and not further exacerbate―the “digital divide” ^(30–32)^.

Importantly, RHM can provide access points to health care for patients with cancer that live in geographically isolated areas or medically underserved regions, both within the United States and globally. However, a significant barrier to successfully deploying RHM systems in rural areas is the lack of internet connectivity ^(14)^. This is especially troubling as cancer mortality rates in rural areas, such as Appalachia, can be significantly higher than in more populated regions ^(33–36)^, making the need for accessible oncology care even more critical. The majority of patients in our BESI-C study live in rural Virginia with inconsistent internet service. While BESI-C does not require an internet connection to collect and locally store data from our wearable and environmental sensors, it is needed for us to “see” our system to assess proper functionality and user engagement and to ensure data files are being properly uploaded into our secure cloud. This remote viewing of data and file upload is essential for our team to analyze data in real-time and ultimately to deliver just-in-time notifications during deployments. Consequently, we currently use a multiprong approach to ensure internet connection, relying on a mobile hot-spot with internet service through a cellular provider (eg, T-Mobile or Verizon) as a first option, and then piggy-backing onto the participant’s local/home WiFi network, if available and with their permission, as a back-up. In reality, we often must deal with limited “viewing” of our data during deployments, and discussions of how to approach this is a frequent and lively point of discussion during team meetings. Nurses can serve as key advocates with legislators and policy makers for enhanced broadband access, thus increasing the reach and impact of RHM.

#### Privacy and data security

Attention to privacy and data security are essential to design and deploy ethical RHM systems ^(30)^. Appropriate investment in team members with experience in health-related research and who are skilled at building secure and compliant data ingest and storage systems (such as cloud-based, relational databases) is essential and should not be underestimated during budget and personnel planning. Ensuring privacy requires transparent informed consent regarding the type of data collected and their ultimate use; reducing or eliminating unnecessary data sources (ie, just because we *can* collect the data does not mean we *should*; asking ourselves questions such as, “are video or audio data *really* needed?”); de-identifying data streams and ensuring secure data transfer and storage; and making it easy for participants to “opt-out” of system monitoring, such as by simply unplugging environmental sensors, giving choice and control to participants about where to place sensors in the home, or removing a wearable. With any collected sensing variable, it is important to ask what features are most important, and critically consider how they can be collected with minimal invasiveness and the most privacy-preservation. For example, in BESI-C we collect audio data to explore possible correlations between ambient sound and pain events. This is obviously potentially very sensitive data, and it is critical this is done in a way to optimally preserve participant privacy. To meet this goal, all audio data are processed locally within the actual environmental sensor node when captured by the microphone and then immediately discarded; only aggregate features of interest, such as the amplitude of sound, versus any intelligible discrete sounds, are transmitted for analysis. Therefore, no conversations can be reconstructed.

#### Participant recruitment and burden

Depending on the specific oncology population under study, recruitment can be extremely difficult and time consuming. For example, there is a large need for research at the end-of-life, but recruiting patients and family caregivers who are coping with terminal, advanced cancer is notoriously challenging, both logistically and ethically ^(37–40)^. RHM systems―particularly in the context of early-stage research, where the direct benefit to patients may be limited―may be viewed as obtrusive or inappropriate at the end-of-life. All RHM technology, regardless of patient group or stage of disease, should be carefully designed to avoid unnecessary participant burden or invasiveness. Thoughtful decisions, made in close collaboration with on-the-ground, front-line clinical partners, are required regarding the realistic and appropriate level of expected active user engagement, such as answering EMAs on wearables or smartphones, and the type, duration, and amount of data collection truly needed to achieve study aims. Optimizing passively collected data can be especially useful for seriously ill cancer populations, but trade-offs between data and burden may still be needed. For example, COVID-19 required a pivot of BESI-C to a completely “contact-less” deployment system that patients and caregivers could set up themselves. Our pre-COVID system (set-up by our study team in a dyad’s home) deployed up to 15 environmental sensors with redundant sensors placed in selected rooms to optimize data collection. However, to minimize participant burden and make system self-installation realistic for patients and caregivers, we reduced this to one environmental sensor placed in up to 4 separate rooms and adjusted our data analysis approach accordingly. Other key lessons learned related to recruitment with BESI-C, in which we recruit patient-caregiver dyads from both a palliative care clinic and a local hospice, include: the need for accurate a priori data regarding average hospice length of stays (being sure to omit outliers from any calculations); developing strong, trusting and mutually beneficial partnerships with all recruitment sites; having a dedicated Clinical Research Coordinator physically (or virtually) present for all clinical or interdisciplinary team meetings to remind busy clinicians about the study and help determine participant eligibility; and considering nested, multiarm studies that allow for flexible recruitment of permutations of various participant groups, such as patients alone; patients and caregivers, and caregivers alone.

#### Personalized and tailored care

Tremendous opportunities exist for nurse-led RHM systems to facilitate personalized and tailored approaches to cancer care. A comprehensive understanding of the patient’s unique symptom experience at home through heterogenous monitoring can ideally inform more specific and effective interventions that, in turn, can help mitigate pain and distress. For example, both passive and active data are collected by BESI-C to comprehensively represent the home environment, the patient’s experience with cancer-related pain, and the caregiver’s experience with the patient’s cancer-related pain. When pain events are recorded by either participant, we are able to capture a holistic snapshot of the pain experience. This will ultimately allow us to build predictive models that could help determine, for example, that for this particular dyad, on Friday afternoons, when the temperature is high, and the caregiver has slept poorly, distress levels are likely to escalate. Armed with this knowledge, we could then deliver a tailored, just-in-time notification that may encourage the caregiver to take a nap or the patient to turn down the room temperature. Relatedly, meaningful data summaries and visualizations generated from RHM systems can promote enhanced shared decision-making between patients and clinicians ^(41–43)^. With BESI-C, we are particularly eager to explore how collected data are best represented to key stakeholders, and exploring how, when, and in what ways to best share data with patients, family caregivers, and clinicians ^(15)^.

#### Rapid technological change

Technology moves fast. In the time that elapses between study conception, proposal development and grant funding, features and functionality of devices have evolved, and certain platforms lose relevance or become obsolete. This challenge may not be as relevant for industry, where agile design processes are embedded into the workflow, and systematically evaluating outcomes is usually not the goal. But for research, obtaining a stable technological platform to compare groups or assess the impact of an intervention over time is critical; this can be especially challenging when commercial devices push out required operating system updates or other changes to their platform. Relatedly, institutional review boards (IRBs) that review health-related protocols are not always equipped or nimble enough to provide investigators with efficient and feasible mechanisms to account for rapidly emerging needs related to data monitoring, storage, and security. Nurse investigators should understand the advantages (ease of use, aesthetics, scalability/availability, and user familiarity) and disadvantages (cost, less control over system design, updates, or changes to platform) of using commercially available devices when designing and deploying RHM systems. It is essential to account for needed device upgrades and changes with grant budgets, timelines, and study design. IRB committees and review panels should ideally include nurse investigators with experience related to RHM research.

#### Family caregiver perspectives

As with any serious illness, cancer affects not only the patient, but the patient’s circle of support. As patients undergo, and recover from, chemotherapy, immunotherapy, surgery, and radiation, family caregivers often play a significant role in supporting the patient, which can take a toll on their own health and well-being (^2,44–46)^. There is much we still do not understand about the cancer caregiving experience, especially in the context of older adults with cancer ^(47)^. RHM systems allow rich opportunities for us to better understand not only the patient experience, but also the experience of those surrounding the patient. RHM systems that collect dyadic data (from patient and family caregiver) or even triadic data (patient, family caregiver, and clinician/or another person) are inherently more complex to design, deploy and interpret but are needed to fully understand the cancer experience and inform holistic and multidimensional interventions. A crucial component of BESI-C is collecting pain event data from both patients *and* family caregivers. EMAs generated on smartwatches worn by both patients and their family caregivers allow participants to report on both their own experience, as well as their perceived partner’s experience. From these data, we are then able to explore how the patient’s experience of pain may influence a caregiver’s mood, activity, and sleep quality and vice versa.

#### Naturalistic data collection

When working with RHM it is important to keep in mind that the data may not be what you think they are. This begins with acknowledging and recognizing the technical limitations of sensor platforms. For example, when we “sense” sleep we are more accurately measuring correlates of sleep, such as motion, EEG signals, or breathing patterns, and then from these proxies we make inferences about sleep. This can be additionally complicated when inferences are drawn from commercially available RHM devices not specifically regulated by the Federal Drug Administration for medical use, and thus with unverified data accuracy ^(4)^. Also, deploying RHM systems in natural settings, such as patient homes (often referred to as “in the wild”), makes it difficult to always know the critical context for those data collection. With BESI-C, we have experienced unusual data from events such as when a localization beacon fell off the top of the refrigerator into a patient’s freezer; a caregiver did not understand they were supposed to wear the smartwatch even when not physically with the patient; a thunderstorm knocked out power to the home for 2 days; a dyad plugged an environmental sensor into an electrical outlet controlled by a light switch; a patient’s watch left on the kitchen counter made heartrate data look alarming. These are just a few examples of how messy sensing data can be when it is generated by real people living real lives. Despite the messiness, sensing data collected from the actual reality in which people experience health has definite advantages, including a truer picture of a patient’s situation that can lead to interventions that are more realistic, feasible, and helpful. Ground truth logs, or structured ways to document and compare what actually happened in the home with the sensing data you receive, are imperative to help contextualize and interpret RHM data.

### Future Directions

The field of RHM is rapidly advancing in scale, scope, and complexity and will increasingly affect health care delivery through the integration of artificial intelligence and machine learning algorithms into the clinical workflow. For nurses to be leaders in addressing the challenges and opportunities outlined above, and to contribute to innovative RHM research, they must be trained to be equal partners in this work (^26,48,49^). This means, in part, offering training opportunities to prepare nurses with the language and baseline skillset to communicate and collaborate effectively with engineers and data scientists, and likewise equipping engineers and data scientists with the equivalent clinical grounding. For example, courses and programs that draw on best practices of interdisciplinary education can equally engage engineers and nurses to teach each other about their respective fields (eg, engineering students could lead sessions related to software programming concepts, sensor design, and networking, while nursing students could teach engineering students about patient assessment, symptom management, and basic principles of pharmacology). These types of cross-pollination educational opportunities ^(50)^―for both students and established professionals―are essential to build the foundations for successful team science and facilitate more nurse-led RHM interventional research.

In conclusion, RHM systems offer novel and innovative approaches to improve the health and well-being of older adults with serious illness. With their clinical background and expertise, nurses are essential to design and deploy RHM systems that are safe, ethical, relevant, and equitable and that can optimally benefit patients, family caregivers, and clinicians. Understanding both the challenges and opportunities related to RHM allows nurses to more actively and confidently engage in this important research and further advance the field.
